# Bioactive Peptides and Depsipeptides with Anticancer Potential: Sources from Marine Animals

**DOI:** 10.3390/md10050963

**Published:** 2012-04-26

**Authors:** Guadalupe-Miroslava Suarez-Jimenez, Armando Burgos-Hernandez, Josafat-Marina Ezquerra-Brauer

**Affiliations:** Department of Research and Food Science Graduate Program, University of Sonora, Apartado Postal 1658, Hermosillo, Sonora, Mexico; Email: msuarez@guayacan.uson.mx (G.-M.S.-J.); aburgos@guayacan.uson.mx (A.B.-H.)

**Keywords:** bioactive peptide, anticancer, antiproliferative, marine compounds

## Abstract

Biologically active compounds with different modes of action, such as, antiproliferative, antioxidant, antimicrotubule, have been isolated from marine sources, specifically algae and cyanobacteria. Recently research has been focused on peptides from marine animal sources, since they have been found as secondary metabolites from sponges, ascidians, tunicates, and mollusks. The structural characteristics of these peptides include various unusual amino acid residues which may be responsible for their bioactivity. Moreover, protein hydrolysates formed by the enzymatic digestion of aquatic and marine by-products are an important source of bioactive peptides. Purified peptides from these sources have been shown to have antioxidant activity and cytotoxic effect on several human cancer cell lines such as HeLa, AGS, and DLD-1. These characteristics imply that the use of peptides from marine sources has potential for the prevention and treatment of cancer, and that they might also be useful as molecular models in anticancer drug research. This review focuses on the latest studies and critical research in this field, and evidences the immense potential of marine animals as bioactive peptide sources.

## 1. Introduction

**Figure 1:**
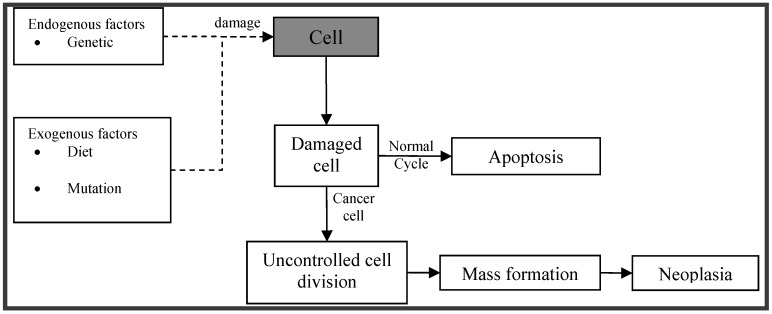
Schematic depiction of pathophysiology of cancer.

Cancer is one of the leading causes of death in the developed world. Cell division is a physiological process that occurs in tissues. Balance between proliferation and programmed cell death is maintained under normal circumstances, usually in the form of apoptosis, by tightly regulating both processes. Certain mutations in DNA lead to cancer by disrupting the programming regulating processes. Carcinogenesis is a process by which normal cells are transformed into cancer cells (Figure 1). It is characterized by a progression of changes at both, cellular and genetic level, that reprogram a cell to undergo uncontrolled division, thus forming a malignant mass (tumor) that can spread to distant locations [1].

Dietary compounds have been isolated and identified in order to contribute to both, good health maintenance and prevention of chronic diseases such as cancer. There has been increased focus on bioactive peptides, which have been defined as “food derived components (naturally occurring or enzymatically generated) that, in addition to their nutritional value exert a physiological effect in the body” [2].

Compounds from marine sources have been reported to have bioactive properties with varying degrees of action [3,4,5], such as anti-tumor, anti-cancer, anti-microtubule, anti-proliferative, anti-hypertensive, cytotoxic, as well as antibiotic properties [6,7,8]. These compounds, that have been isolated from marine sources are of varying chemical nature including phenols, alkaloids, terpenoids, polyesters, and other secondary metabolites which are present in sponges, bacteria, dinoflagellate, and seaweed [9]. Since biodiversity of the marine environment far exceeds that of the terrestrial environment, research on the use of marine natural products as pharmaceutical agents has been steadily increasing. Throughout evolution, marine organisms have developed into very refined physiological and biochemical systems; therefore, these organisms have developed unique adaptation strategies that enable them to survive in dark, cold, and highly pressurized environments. On the other hand, there is an intense competition for survival among the wide variety of species. All these species have developed chemical means to defend against predation, overgrowth by competing species, or conversely, to subdue motile prey species for ingestion. Also, secondary metabolites, which are produced by marine invertebrates and bacteria, have yielded medicinal products such as novel anti-inflammatory, anti-cancer, and antibiotics agents [10].

Food-derived bioactive peptides represent one source of health-enhancing components. These peptides may be released during gastrointestinal digestion or food processing from a multitude of plant and animal proteins, especially milk, soy, and fish proteins [11]. Recently, there has been an increment in the number of studies focused on marine bioactive peptides. Many bioactive peptides and depsipeptides with anticancer potential have been extracted from various marine animals like tunicates, sponges, soft corals, sea hares, nudibranchs, bryozoans, sea slugs, and other marine organisms [12,13,14]. There is an extensive group of peptides and depsipeptides extracted from marine animals, however, this review focuses on the most studied that have achieved clinical trials and furthermore some that are commercially available such Aplidine [15]. Biologically active peptides obtained from marine animal species are considered to have diverse activities, including opioid agonistic, mineral binding, immunomodulatory, antimicrobial, antioxidant, antithrombotic, hypocholesterolemic, and antihypertensive actions [16]. By modulating and improving physiological functions, bioactive peptides may provide new therapeutic applications for the prevention and/or treatment of chronic diseases. As components of diverse marine species with certain health claims, bioactive peptides are of particular pharmaceutical interest [9].

All substances sold as drugs in the United States must be approved by the federal Food and Drug Administration. This approval process requires a series of phased drug trials. The first phases involve *in vitro* and animal testing. If no adverse side effects are observed and significant ameliorative effects are found, testing on human subjects is undertaken. This process may take years because of the need to search for long-term side effects and to optimize methods for drug administration and dosage [17].

This review compiles the most relevant studies performed in order to comply with development of peptides and depsipeptides derived from marine animals as anticancer drugs. With the latest increase in peptide research, the purpose of this review is to facilitate discussion on this issue since marine peptides are one of the recent perspectives in the development of new compounds for further drugs and therapeutic use in the treatment of cancer. Bioactive peptides and depsipeptides, most currently studied from animal marine species with anticancer potential and which have reached clinical trials, have therefore been examined.

## 2. Sources of Bioactive Marine Peptides

**Figure 2:**
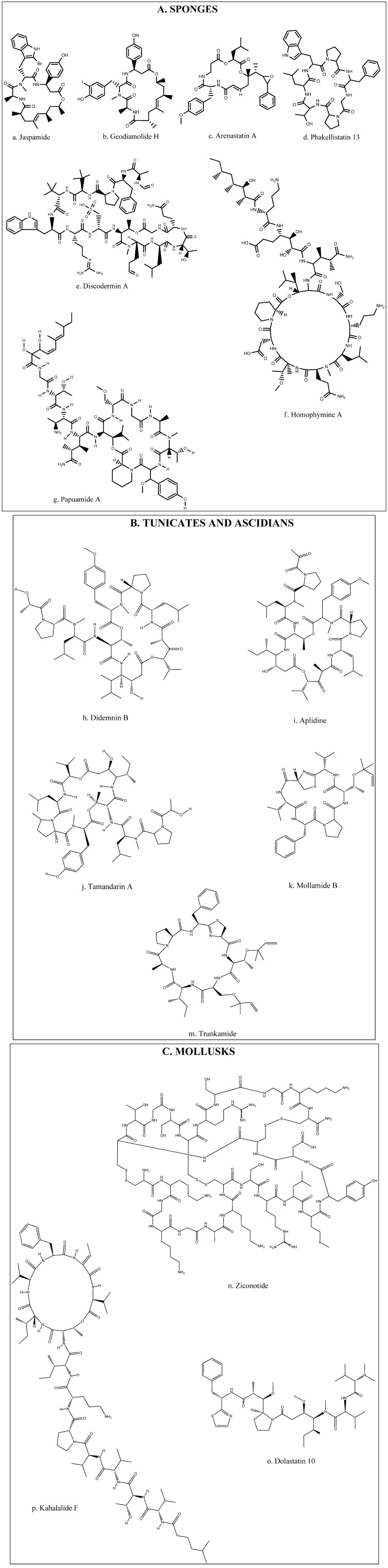
Chemical structures of bioactive peptides and depsipeptides from marine animal sources: (**A**) Sponges; (**B**) Tunicates and Ascidians and (**C**) Mollusks.

A diversity of peptides with bioactivity has been mainly extracted from various marine animals such as tunicates, sponges, and mollusks. This extensive group of bioactive peptides which have been reported in recent studies includes compounds such as Stylisin from Jamaican sponge *Stylissa caribica* [18], Papuamides from sponge of the genus Melophlus collected in the Solomon Islands [19]. Many of these compounds have been isolated, characterized, synthesized and further modified for the development of analogs in order to improve their activities [20,21,22,23]. However, among these bioactive peptides and depsipeptides, several have been studied in depth, and even have been taken to clinical study levels (Table 1). Many of these compounds have biological activities and hence have potential beneficial uses in health promotion or disease treatment [3,6]. Recently, much attention has been paid to discover the structural, compositional, and sequential properties of bioactive peptides from marine sources. Figure 2 illustrates the chemical structures of the most prevalent bioactive peptides and depsipeptides obtained from marine animals such as sponges, ascidians, tunicates and mollusks. Three methods have been used to produce marine bioactive peptides; solvent extraction, enzymatic hydrolysis, and microbial fermentation of marine proteins. However, particularly in food and pharmaceutical industries, the enzymatic hydrolysis method is preferred on account of the lack of residual organic solvents or toxic chemicals in the products [11].

**Table 1:** Marine animal sources of bioactive peptides with anticancer potential.

Compound	Source	Organism	Bioactivity	Reference
**Aplidine**	Ascidian	*Aplidium albicans*	Antitumor Anti leukemic	[24,25]
**Arenastatin A**	Sponge	*Dysidea arenaria*	Antitubulin	[26,27,28]
**Aurilide**	Tunicate	*Dolabella auricularia*	Antitumor	[29,30]
**Didemnin**	Tunicate	*Trididemnum* sp.	Antitumor	[3,31]
**Dolastatin**	Mollusk	*Dolabella auricularia*	Antineoplastic	[32]
**Geodiamolide H**	Sponge	*Geodia* sp.	Antiprolfierative	[28,33]
**Homophymines**	Sponge	*Homophymia* sp.	Antitumor	[34]
**Jaspamide**	Sponge	*Jaspis* sp. *Hemiastrrella* sp.	Antiproliferative	[35,36]
**Kahalalide F**	Mollusk	*Elysia rufescens*, *Spisula polynyma*	Antitubulin	[28]
**Keenamide A**	Mollusk	*Pleurobranchus forskalii*	Antitumor	[37]
**Mollamide**	Ascidian	*Didemnum molle*	Antiproliferative	[30,38]
**Phakellistatins**	Sponge	*Phakellia carteri*	Antiproliferative	[30,39]
**Tamandarins A and B**	Ascidian	*Didemnum* sp.	Antitumor	[30,40]
**Trunkamide A**	Ascidian	*Lissoclinum* sp.	Antitumor	[30,41]

### 2.1. Sponges

Approximately 10,000 sponges have been described worldwide and most of them live in marine environments [42,43]. A range of bioactive compounds has been found in about 11 sponge genera. Three of these genera (Haliclona, Petrosia, and Discodemia) produce influential anti-cancer and anti-inflammatory agents [44]. There are a number of research studies on bioactive peptides from sponges, mostly cyclodepsipeptides, which are secondary metabolites with unusual amino acids and non-amino acid moieties. These compounds possess a wide spectrum of biological activities; however, it is difficult to isolate them in sufficient quantity for pharmacological testing [30].

Jaspamide is a cyclic depsipeptide isolated from sponges of the genus *Jaspis* and *Hemiastrella*. It possess a 15-carbon macrocyclic ring containing three amino acid residues (Figure 2a) and has proved to be a bioactive compound inducing apoptosis in HL-60 human promyelocytic leukemia cell line [7,35,45], and Jurkat T cells [46]. Nine new cyclodepsipeptides, *Homophymines*, B–E, and A1–E1, isolated from the sponge *Homophymia* sp. have shown very potent cytotoxic activity with IC_50_ values in the nM range. This activity has been reported against several human cancer cell lines [28,34] with moderate selectivity against human prostate (PC3) and ovarian (OV3) carcinoma. *Homophymines* A1–E1, which possesses the 4-amino-6-carbamoyl-2,3-dihydroxyhexanoic acid residue (Figure 2f), exerts stronger potency than the corresponding A–E compounds which possess the same residue present in its carboxy form [34].

*Geodiamolide* H (Figure 2b) isolated from a Brazilian sponge *Geodia corticostylifera* have demonstrated antiproliferative activity against breast cancer cells by altering the actin cytoskeleton [33]. *Discodermins* tetradecapeptides are another group of cytotoxic peptides obtained from sponges of the genus *Discodermia* sp. containing 13–14 known and rare amino acids as a chain, with a macrocyclic ring constituted by lactonization of a threonine unit with the carboxy terminal (Figure 2e). *Discodermins* A–H were tested against A549 human lung cell line and P388 murine leukemia cells, all showing cytotoxicity [3].

*Arenastatin* A (Figure 2c) is a cyclodepsipeptide isolated from *Dysidia arenaria* that have demonstrated a potent cytotoxicity against KB cells with an IC_50_ of 5 pg/mL [3]. *Papuamides* A–D isolated from sponges of the genus *Theonella*, are the first marine-derived peptides reported to contain 3-hydroxyleucine and homoproline residues and a 2,3-dihydroxy-2,6,8-trimethyldeca-(4*Z*,6*E*)-dienoic acid moiety, *N*-linked to a terminal glycine residue (Figure 2g). It has also been discovered that *Papuamides* A and B inhibited the infection of human *T*-lymphoblastoid cells by HIV-1 *in vitro* [3,47].

*Phakellistatins* isolated from the Western Indian Ocean sponge *Phalkellia carteri* inhibit leukemia cell growth [39]. Another related compound, *Phakellistatin* 13 (Figure 2d) from sponge *Phakellia fusca* was cytotoxic against the human hepatoma BEL-7404 cell line with an ED_50_ < 10^−2^ μg/mL. Synthetic specimens of Phakellistatin were found to be chemically but not biologically (cancer cell lines) identical to the natural products. The reason might be a conformational difference, especially around the proline residue [30,48].

### 2.2. Tunicates and Ascidians

Bioactive peptides with novel structures have also been shown in ascidians. Sack-like sea squirts inhabiting the sea floor, produce a complex anti-tumor compound which is, hundreds to thousands of times more influential than any cancer concoction now in use [10]. One of these potent compounds is *Didemnin*, isolated at first from the Caribbean tunicate *Trididemnum solidum* but later obtained from other species of the same genus [3,49]. Among these compounds, *Didemnin* B (Figure 2h) has the most potent antitumor activity and also has showed antiproliferative activity against human prostatic cancer cell lines [3,31]. *Didemnin* B inhibits the synthesis of RNA, DNA and proteins [50]. Substantial evidence of activity in preclinical models with dose-dependent and tolerable toxicity profiles led to phase I clinical trials, making this peptide the first marine natural product to be evaluated in clinical trials [51,52]. The toxicity profile of *Didemnin* B was quite similar across the trials, with dose-dependent nausea and vomiting as the most commonly reported side effects. Phase II trials using *Didemnin* B at the recommended doses were inefficient, while trials using more aggressive regimens resulted in higher levels of toxicity, including cardiotoxicity [53,54,55].

*Aplidine* (Figure 2i) is a cyclodepsipeptide isolated from the tunicate *Aplidium albicans*, which has been shown to have anticancer activity against a variety of human cancer cell lines, such as breast, melanoma and lung cancers [28,56], which appear to be sensitive to low concentrations of this compound. Aplidine’s mode of action involves several pathways, including cell cycle arrest and inhibition of protein synthesis, thus inducing apoptosis of cancer cells [57]. Furthermore, *Aplidine* possesses a unique and differential mechanism of cytotoxicity which involves the inhibition of ornithine descarboxylase, an enzyme that is critical in the process of tumor formation and growth [24]. *Aplidine* also inhibits the expression of the vascular endothelial growth factor gene, having antiangiogenic effects [25]. *Aplidine*, was well tolerated with minor toxicity in finished Phase I clinical trials with the most common side effects being asthenia, nausea, vomiting and transient transaminitis, but not inducing hematological toxicity, mucositis or alopecia [56,58,59]. Neuromuscular toxicity with the elevation of creatine phosphokinase levels has been dose limited, but seemed to be readily reversible with oral carnitine [56]. *Aplidine* has shown antitumor activity in phase I trials [56,58], and has already undergone active phase II studies in solid tumors [60,61,62].

*Tamandarins* A (Figure 2j) and B are also cytotoxic depsipeptides from a marine ascidian of the family Didemnidae, which was evaluated against various human cancer cell lines [30,40]. *Mollamide* is a cyclodepsipeptide obtained from the ascidian *Didemnum molle*, and it has shown cytotoxicity against a range of cell lines with IC_50_ values of 1 μg/mL toward P388 murine leukemia line and 2.5 μg/mL against A549 human lung carcinoma and HT29 human colon carcinoma [30,38]. *Trunkamide* A is a cyclopeptide with a tiazoline ring similar to *Mollamide* (Figure 2k,m) obtained from ascidians of the genus *Lissoclinum*, where antitumor activity under preclinical trials has been demonstrated [30,41].

### 2.3. Mollusks

Mollusks are species that have a wide range of uses in pharmacology. Sea hare, a shelled organism, produces bioactive metabolites used in the treatment of cancerous tumors [10]. *Ziconotide* is a 25 amino acid peptide with three disulphide bonds (Figure 2n); and is present in the venom of the predatory Indo-Pacific marine mollusk, *Conus magus.* It possesses remarkable analgesic activity, which has proved to be 1000 times more active than morphine in animal models of nociceptic pain [63]. Cone snails belonging to the genus *Conus* are a valuable source of active peptides named conotoxins. They consist of a mixture of peptides with short chains of amino acids (8–35) rich in disulfide. Studies have postulated that these peptides could be of interest in the treatment of cancer [3,64].

*Dolastatins* is a family of cytotoxic peptides isolated from the mollusk *Dollabella auricularia*, where the linear pentapeptide *Dolastatin* 10 (Figure 2o) and the depsipeptide *Dolastatin* 15 have had the most promising antiproliferative activity reported [65,66]. *Dolastatin* 10 is an antineoplastic substance proven against several cancer cell lines [67] and has been evaluated in various phase I clinical trials reporting good tolerability and identifying myelosuppression as the dose limiting toxicity. Other side effects observed were peripheral sensory neuropathies, pain, swelling, and erythema at the injection site [67,68]. Complexity and low yield of chemical synthesis of dolastatins, together with low water solubility, have been significant obstacles to broader clinical evaluation, triggering the development of analog compounds [69,70].

A 60-kDa protein, Bursatellanin-P, was purified from the purple ink of the sea hare *Bursatella leachii* showing anti-HIV activity [71]. *Keenamide A* is a cytotoxic cyclic hexapeptide isolated from the mollusk *Pleurobranchus forskalii*, which elicits antitumor activity via unknown mechanisms. This compound exhibited significant activity against the P388, A549, MEL-20 and HT-29 tumor cell lines [37].

Kahalalides is a family of peptides isolated from the sacoglossan mollusk *Elysia rufescens*. Among these, Kahalalide F is a dehydroamino-butyric acid-containing peptide (Figure 2p) which is known to exhibit interesting antitumor activity [72]. Kahalalide F has shown *in vitro* and *in vivo* selectivity for prostate-derived cell lines and tumors [73,74]. It has been observed that Kahalalide F induces disturbances in lysosomal function that might lead to intracellular acidification and cell death. These results suggest that cells with high lysosomal activity, such as prostate cancer cells, would be a suitable tumor type to use to explore the activity of this peptide [72]. In phase I clinical trials, Kahalalide F exhibited clinical benefits in treated patients and low toxicity with few side effects restricted to fatigue, headache, vomiting, and pruritus limited to the hands. Since hematological toxicities have not been observed, Kahalalide F results show suitability for trials in combination with other anticancer agents [75]. There is evidence that suggests that Kahalalide F may be active against other tumor types and deserves further clinical testing either as a single agent or in combination [75]. Currently, this agent is undergoing phase II clinical trials for the treatment of lung and prostate cancers, and melanoma [76].

### 2.4. Marine Protein Hydrolysates

In recent years, there has been a considerable amount of research focused on the liberation of bioactive peptides encrypted within food proteins, and towards the use of peptides as functional food ingredients that promote health maintenance or as potential drugs for the treatment of chronic diseases. Interestingly, within the parent protein sequence, the peptides are inactive and thus must be released to exert an effect. These bioactive peptides are usually 2–20 amino acid residues in length: however, some have been reported to be longer than 20 amino acid residues [77].

Protein hydrolysis is the method used to obtain peptides from food protein sources with different biological activities, such as antioxidant, antihypertensive, antimicrobial and antiproliferative. It consists of breaking the peptide bond and subsequent generation of smaller peptides or free amino acids, if an adequate control of the hydrolysis is achieved [78]. The protein hydrolysis method most commonly used is enzymatic hydrolysis, since alkaline hydrolysis is not frequently used due to the racemization or destruction of certain amino acids at high pH [79]. On the other hand, the acid method has the disadvantage that tryptophan is completely destroyed, while serine and threonine are destroyed by 5–10% and asparagine and glutamine are hydrolyzed to their corresponding acids [80].

Enzymatic hydrolysis is carried out under controlled pH and temperature conditions that reduce the formation of undesirable products [78]. Several enzymes are used to obtain hydrolysates, among which are the digestive and microbial proteases, including alcalase, trypsin, pepsin, chymotrypsin, pancreatin, pepsin, and thermolysin, among others [81]. Moreover, studies have demonstrated that enzymatic hydrolysis most likely increases the antioxidative activity of the resulting hydrolysate via the enhancement of radical scavenging activity [82].

Fish is an important source of protein worldwide; additionally, fish proteins offer huge potential as novel sources of bioactive peptides. Hydrolysates of several marine proteins have been assayed for various bioactivities. Peptides present in protein hydrolysates have biological activities depending on their molecular weights and amino acid sequences. Crude hydrolysates are subsequently fractionated to separate individual peptides using different techniques, mainly reverse phase high performance liquid chromatography (RP-HPLC) or gel permeation chromatography [4,13,83].

Enzymatic hydrolysis of food proteins is considered an efficient way to recover potent bioactive peptides, since several peptides obtained by this process have different bioactivities and this may represent a potential approach to anticancer drugs. Up to now, bioactive peptides with potential anticancer exhibiting antioxidant and antiproliferative effects have been found in the hydrolysates of marine proteins [84,85,86,87] (Table 2).

**Table 2:** Bioactivity of peptides from marine protein enzymatic hydrolysates with anticancer potential.

Source	Enzyme	Amino Acid Sequence	Bioactivity	Reference
**Alaska pollack collagen (*Theragra chalcogramma*)**	Trypsin and Flavourzyme	nd	Antioxidant *in vitro*	[88]
**Croaker muscle (*Otolithes ruber*)**	Pepsin, followed by Trypsin + αChymotrypsin	GNRGFACRHA	Antioxidant *in vitro*	[89]
**Flyingfish (*Exocoetus volitans*)**	Trypsin	nd	Antioxidant Antiproliferative for Hep G2	[14]
**Flying squid skin gelatin (*Ommastrephes batramii*)**	Pepsin, followed by Trypsin + αChymotrypsin	nd	Antioxidant *in vitro*	[90]
**Horse mackerel muscle (*Magalapsis cordyla*)**	Pepsin, followed by Trypsin + αChymotrypsin	NHRYDR	Antioxidant *in vitro*	[89]
**Jellyfish umbrella collagen (*Rhopilema esculentum*)**	Trypsin and Flavourzyme	nd	Antioxidant	[88]
**Jumbo flying squid skin gelatin (*Dosidicus gigas*)**	Esperase and Alcalase	nd	Antioxidant *in vitro* Antiproliferative/Cytotoxic on MCF-7 and U87 cells	[82,91]
**Oyster (*Crassostrea gigas*)**	Protease from *Bacillus* sp. SM98011	nd	Antitumor in BALB/c mice	[4]
**Smooth hound (*Mustelus mustelus*)**	LMW alkaline protease	nd	Antioxidant *in vitro*	[92]
**Solitary tunicate (*Styela clava*)**	Alcalase	nd	Antioxidant *in vitro* Antiproliferative on AGS, DLD-1, and HeLa cells	[13]
**Threadfin bream (*Nemipterus japonicas*)**	Trypsin	nd	Antioxidant Antiproliferative on HepG2	[14]
**Tilapia (*Oreochromis niloticus*)**	Cryotin, Flavourzyme, Alcalase	nd	Antioxidant *in vitro*	[93,94]
**Tuna dark muscle byproduct (*Thunnus tonggol*)**	Papain and Protease XXIII	LPHVLTPEAGAT PTAEGGVYMVT	Antiproliferative on MCF7 cells	[83]
**Tuna skin gelatin (*Thunnus* spp.)**	Alcalase	nd	Antioxidant *in vitro*	[82]

nd = not determined.

#### By-Products from Processing Hydrolysates

The most common definition of by-products is that referring to all the raw material remaining after the production of the main products. The general understanding of by-products, when considering round fish such as cod, is that the main body flesh (constituting the fillets) is considered to be the main product, but the head, backbones, trimmings (cut-offs), skin and guts constitute what is generally thought as by-products [95]. The definition of rest raw materials in the fish industry varies with fish species as well as with the harvesting and processing methods used [96].

A major issue for food producers is the discarding of by-products from food processing. Adding value to waste streams is very appealing to food producers, as the by-products are usually incorporated into low economic value products such as animal feed.

Bioactive peptides from various marine enzymatically hydrolyzed by-products such as fish bones [97], shrimp waste [98], tuna head [99], have been identified. Hydrolyzed protein from the viscera of mackerel was used to obtain bioactive peptides [89]. Also, sardinelle by-product hydrolysates have been a good source of peptides with high antioxidant activity [100]. There is a large number of studies on the enzymatic hydrolysis of collagen or gelatin used for the production of bioactive peptides. Among these, squid and tuna skin gelatin hydrolysates, enzymatically produced, have shown antioxidant activity measured by the Fe reducing capacity (FRAP) and the ABTS radical scavenging methods [82].

## 3. Anticancer Activities of Marine Animal Peptides

Bioactive peptides usually contain 2–20 amino acid residues and their activities are based on their amino acid composition and sequence. These peptides are reported to be involved in various biological functions such as, antioxidant, antiproliferative, antitubulin and cytotoxic activities [16,101]. These activities could confer anticancer potential, which will give a use in cancer therapy.

### 3.1. Antioxidative Activity

Antioxidants are known to be beneficial to human health as they may protect the body against molecules known as reactive oxygen species (ROS). ROS can attack membrane lipids, protein, and DNA. This consecutively can be a causative factor in many diseases such as cancer. As ROS are involved in cancer development, compounds with high ROS reduction activity are likely to be able to prevent cancer incidence, since the oxidative stress inhibition leads to reduced genetic alteration such as mutation and chromosomal rearrangements which play a vital role in the initiation of carcinogenesis [13].

Antioxidant peptides have been found in numerous foodstuffs including algae protein waste [102], milk [103] and enzymatically produced protein hydrolysates [84,86,104,105]. Among these, numerous fish protein hydrolysates (from sources such as Tilapia) have demonstrated antioxidant potential. They have significant ability to scavenge ROS and reduce ferric ions [106]. Hydrolysates from mackerel muscle obtained with Protease N, contain peptides with antioxidant activity *in vitro*. The antioxidant activity is measured by the peptide’s capacity to scavenge the free radical α,α-diphenyl-β-picrylhydrazil (DPPH) and reduce Fe^3+^ to Fe^2+^ [107]. This antioxidant potential is similar to that reported for protein hydrolysates from other sources, such as casein enzymatic hydrolysate, which exhibits significant ability to scavenge ROS [108]. Also soy and wheat protein hydrolysates showed strong capacity to scavenge DPPH [109].

Hydrolysates of several skin gelatins such as flying squid (*Ommastrephes batramii*) [90], tuna (*Thunnus* spp.) and jumbo flying squid (*Dosidicus gigas*) [82,110] have been shown to possess antioxidant activity. Gelatin peptides mainly contain hydrophobic amino acids and abundance of these amino acids favors a high emulsifying ability to hydrophilic-hydrophobic partitioning in the peptide sequence [84]. In addition, specific amino acid arrangements with their abundance of Gly, Pro and Hyp, merit special consideration, as the content of Pro residues has a scavenging effect on radicals and the percentage of hydroxylation seems to be related to the antioxidant properties as measured by FRAP [82]. Hence, marine gelatin derived peptides are expected to exert high antioxidant effects among other antioxidant peptide sequences [82,84]. Therefore, marine-derived bioactive peptides with antioxidative properties may have great potential for use as nutraceuticals and pharmaceuticals and as a substitute for synthetic antioxidants.

Muscle hydrolysates of horse mackerel (*Magalapsis cordyla*) and croaker (*Otolithes ruber*) [89], *Nemipterus japonicas* and *Exocoetus volitans* [14] have been shown to have an ability to scavenge free radicals and reactive oxygen species, showing antioxidant activity. Protein hydrolysates obtained from Channel catfish (*Ictalurus punctatus*) protein isolates [104] and from jellyfish (*Rhopilema esculentum*) umbrella collagen [105] have shown antioxidant activity as determined by different methods.

Flying squid gelatin hydrolysate (enzymatically obtained) showed high antioxidant ability. At concentrations of 16 and 12 mg/mL, the hydrolysate showed a superior ability to scavenge DPPH free radicals than BHA and α-tocopherol, respectively. This hydrolysate was found to be rich in antioxidant amino acids including tyrosine, histidine, proline, alanine, and leucine. Furthermore, it appeared that hydrolysate fractions having a molecular weight ranging from 383 to 1492 Da, might be responsible for its antioxidant activity. Moreover, the size (usually lower molecular weight) and the amino acid composition were found to be strongly correlated to their antioxidant activity [90]. The mechanisms of action of peptides as antioxidants is not clearly known, but its activity has been attributed to certain amino acid sequences, that include some aromatic amino acids and histidine [111]. High amounts of histidine and some hydrophobic amino acids are associated with antioxidant potency [112]. The activity of histidine-containing peptides is thought to be connected to a hydrogen-donating ability, lipid peroxyradical trapping, and/or the metal ion chelating ability of the imidazole group [113]. The addition of a leucine or proline residue to the *N*-terminus of a histidine–histidine dipeptide would enhance antioxidant activity. The hydrophobicity of the peptide also appears to be an important factor for its antioxidant activity due to an increased accessibility to hydrophobic targets [114].

### 3.2. Antiproliferative Activity

*Didemnin* depsipeptides are cytotoxic to cancer cell lines by inhibiting protein synthesis *in vitro* [115]. It is suggested that protein synthesis may be inhibited by the binding of *Didemnins* to ribosome-EF-1α complex, since there is a correlation between inhibiting protein synthesis in cell lysates and in human adenocarcinoma MCF-7 cells [116]. Studies with Jaspamide in HL-60 human leukemia cell line revealed that nanomolar concentrations of this depsipeptide induced inhibition of cell proliferation and increased polynuclear cells [35].

*Cryptophycin*-52, a member of the family of the marine depsipeptides Cryptophycins, produced by total chemical synthesis, showed antitumor activity at picomolar concentrations. This compound was shown to inhibit cancer cell proliferation by stabilizing spindle microtubules, binding tightly and non-covalently to a single high-affinity site on tubulin, while also inducing a conformational change in the tubulin molecule [7,117].

Peptides and amino acids from several dietary proteins have been reported to show antitumor or antiproliferative activities, most of them from vegetal sources [118,119,120,121]. However, the antiproliferative activities of marine proteins have been barely studied. Hydrolysates from three blue whiting, three cod, three plaice and one salmon were identified as significant growth inhibitors on MCF-7/6 and MDA-MB-231 cell lines. Composition analysis evidenced they contained a complex mixture of free amino acids and peptides of various sizes ranging up to 7 kDa [85]. An enzymatic protein hydrolysate of oyster inhibited the growth of transplantable sarcoma-S180 in a dose-dependent manner in BALB/c mice, showing strong immunostimulating effects [4]. The antitumor drug cyclophosphamide, which possesses a high tumor inhibitory rate, was also shown to have a strong immunosuppressive effect [122]. In contrast, oyster hydrolysates inhibited tumor growth by improving the immune function in S108-bearing mice, which suggests a potential use in tumor therapy [4]. An enzymatic hydrolysate from jumbo squid skin gelatin showed cytotoxic effect against MCF-7 and U87 cell lines, with IC_50_ values of 0.13 and 0.10 mg/mL, respectively [91]. Solitary tunicate hydrolysate exhibited strong antioxidant activity, including DPPH, ABTS, H_2_O_2_, and OH radical scavenging activities. Moreover, this hydrolysate also showed potent anticancer activity against AGS, DLD-1, and HeLa cancer cells. However, the anticancer activities of these fractions (IC_50_ 577.1–1240.0 μg/mL) were much lower than that of commercial standards such as Paclitaxel (IC_50_ 2.2–24.6 μg/mL) and 5-Fluorouracyl (IC_50_ 3.4–34.5 μg/mL) [13].

Peptide fraction of *Nemipterus japonicas* and *Exocoetus volitans* hydrolysates exerted significant antiproliferative effect on human hepatocellular liver carcinoma cell lines (Hep G2) with IC_50_ values 48.5 mg/mL and 21.6 mg/mL, respectively. Moreover, these fractions did not show any cytotoxicity effect for Vero (kidney epithelial cells of the African Green Monkey) cell lines [14]. Peptides isolated from enzymatic hydrolysate of tuna dark muscle by-product show a dose-dependent inhibition effect of the MCF-7 cells with IC_50_ values of 8.1 and 8.8 μM [83]. These results showed that tuna dark muscle by-product might be a good source to produce antiproliferative peptides which may be useful in therapy as agents with high pharmaceutical value.

Isolation and identification of the specific peptide sequences of peptides that are responsible for the antioxidative and anticancer effects also should be carried out. It may be assumed that the low molecular weight peptides have greater molecular mobility and diffusivity than the high molecular weight peptides, which appears to improve interactions with cancer cell components and enhances anticancer activity [13]. Although a study on the mechanism of action revealed that modulation of hydrophobicity of peptides plays a crucial role against cancer cells [123]. However, studies on the effects of the antiproliferative peptides on cell cycle of normal and transformed cells, on the structure of the bioactive peptides, and *in vivo* studies of these activities, need to be further investigated.

## 4. Pharmacological Application and New Perspectives of Bioactive Peptides

Currently the number of natural products is increasing; however, very few compounds have reached the market. A limited number of identified peptides found in marine animals are in preclinical trials and some of them have made it to different phases of clinical trials to prove their potential as antitumor drugs. *Cemadotin*, a peptide obtained from sea slug and *Aplidine*, a potent apoptosis inducer depsipeptide isolated from tunicate *Aplidin albicans*, are under phase II clinical trials [61,62]. Kahalalide F which has shown antitumor activity [72] has recently undergone phase III clinical trials for the treatment of lung and prostate cancers along with melanoma [76].

Limited research on bioactive marine animal peptides may be due to the lack of sufficient quantities of the compounds, problems in accessing the source of the samples, difficulties in isolation and purification procedures as well as to ecological considerations. Moreover, chemical synthesis of these peptides plays an important role in structure determination. This is challenging since the synthesis of the required amounts of the compound might constitute a problem, and moreover it has been demonstrated that some conformational issues are determinant in the bioactivity of these molecules.

Peptides produced by enzymatic hydrolysis of marine proteins are an alternative source of bioactive compounds with anticancer potential, since they have shown antioxidant and antiproliferative activities. However, *in vivo* studies are needed in order to achieve complete anticancer drug development. The use of specific enzymes enables the selection of rupture sites in the protein sequence that could be determinant for peptide bioactivity. However, there is a need for further research in order to elucidate the bioactive peptide structure, to determine its mode of action, and to determine the way it interacts with the cancer cell cycle.

Increasing use of genomics combined with biosynthesis might represent a strategy for the production of natural marine peptides. An alternative would be that the advances in the field of genomics, proteomics and metabolomics could have a high impact on the identification and production of peptides as antitumor agents. Finding the coding sequence of DNA that codifies for bioactive peptide will be a significant achievement for the production of these compounds.

## 5. Conclusions

Finding a cure for cancer is one of the greatest actual challenges for pharmacology and medicine. There is an extensive research effort aimed at obtaining efficient compounds of natural origin. Most of the marine peptides subjected to clinical trials are secondary metabolites from animals, but there exists a widely unexplored field in marine protein hydrolysates.

Studies on peptides obtained from protein hydrolysates, have shown that these molecules have antioxidant, antiproliferative, and antimutagenic activities which could confer on them anticancer potential; however, more research on the mode of action on the cell cycle or apoptosis of cancer cell lines is necessary. Nevertheless, there is a need for scaled-up production of these compounds, which could be achieved by utilization of marine byproducts.

## Supplementary Files

Supplementary File 1: ZIP-Document (ZIP, 46 KB)
